# Sex Hormones Related Ocular Dryness in Breast Cancer Women

**DOI:** 10.3390/jcm10122620

**Published:** 2021-06-14

**Authors:** Antonella Grasso, Antonio Di Zazzo, Giuseppe Giannaccare, Jaemyoung Sung, Takenori Inomata, Kendrick Co Shih, Alessandra Micera, Daniele Gaudenzi, Sara Spelta, Maria Angela Romeo, Paolo Orsaria, Marco Coassin, Vittorio Altomare

**Affiliations:** 1 Breast Unit, University Campus Bio-Medico, 00128 Rome, Italy; a.grasso@unicampus.it (A.G.); p.orsaria@unicampus.it (P.O.); v.altomare@unicampus.it (V.A.); 2 Ophthalmology Operative Complex Unit, University Campus Bio-Medico, 00128 Rome, Italy; d.gaudenzi@unicampus.it (D.G.); s.spelta@unicampus.it (S.S.); m.coassin@unicampus.it (M.C.); 3 Department of Ophthalmology, University Magna Graecia of Catanzaro, 88100 Catanzaro, Italy; giuseppe.giannaccare@unicz.it; 4 Department of Ophthalmology, School of Medicine, Juntendo University, 1130033 Tokyo, Japan; jsung1@usf.edu (J.S.); tinoma@juntendo.ac.jp (T.I.); 5 Department of Ophthalmology, Li Ka Shing Faculty of Medicine, The University of Hong Kong, Hong Kong; kcshih@hku.hk; 6 Research and Development Laboratory for Biochemical, Molecular and Cellular Applications in Ophthalmological Sciences, IRCCS–Fondazione Bietti, 00198 Rome, Italy; alessandra.micera@fondazionebietti.it; 7 School of Medicine, Humanitas University, 20089 Milan, Italy; romeo.mariaangela@gmail.com

**Keywords:** dry eye syndrome, breast cancer, sex hormones, ocular surface equilibrium

## Abstract

Background: Dry eye syndrome (DES) is strictly connected to systemic and topical sex hormones. Breast cancer treatment, the subsequent hormonal therapy, the subsequent hyperandrogenism and the early sudden menopause, may be responsible for ocular surface system failure and its clinical manifestation as dry eye disease. This local dryness is part of the breast cancer iatrogenic dryness, which affects overall mucosal tissue in the fragile population of those with breast cancer. Methods: A literature review regarding the role of sex hormone changes and systemic hormonal replacement treatments (SHRT) in DES available on PubMed and Web of Science was made without any restriction of language. Results: Androgens exert their role on the ocular surface supporting meibomian gland function and exerting a pro-sebaceous effect. Estrogen seems to show a pro/inflammatory role on the ocular surface, while SHRT effects on dry eye are still not well defined, determining apparently contradictory consequences on the ocular surface homeostasis. The role of sex hormones on dry eye pathogenesis is most likely the result of a strict crosstalk between the protective androgens effects and the androgen-modulating effects of estrogens on the meibomian glands. Conclusions: Patients with a pathological or iatrogenic hormonal imbalance, such as in the case of breast cancer, should be assessed for dry eye disease, as well as systemic dryness, in order to restore their social and personal quality of life.

## 1. Introduction

Dry eye syndrome is the most common ocular surface disorder, affecting one in five people, with increased prevalence in women than men [1].

The epidemiological sexual disparity of dry eye prevalence suggests sex hormone changes may influence the composition of the tear film as well as the function of different ocular surface structures and components [2,3,4,5,6]. Sex hormones, estrogen, progesterone, and testosterone, are known to play important and different roles in ocular surface homeostasis. The ocular surface, including lacrimal glands, meibomian glands, conjunctiva, and corneal epithelium, contains receptors for both estrogens and androgens [7]. As such, sex hormones are vital in the production of the main components of our tear film, including the aqueous layer, lipid, and mucin. The absolute hormone levels, their fluctuations, and changes in hormone receptor (HR) responsiveness are all important factors in determining ocular surface stability.

Systemic sex hormones and their local receptor expression levels may up- or downregulate themselves during the physiological fluctuation of menstrual cycles or menopause [8,9,10,11].

Moreover, in mucosal tissue, the protective mucin barrier appears to be largely influenced by local and circulating sex hormone levels, by modulating key mucin components [3,6,12]. This apical epithelial barrier is constituted by transmembrane mucins, carbohydrate-binding proteins named galectins and soluble mucins, which are highly expressed by the ocular surface [13,14,15,16].

Therefore, the effects of sex hormones are clinically seen in women after breast cancer surgery. In fact, those patients require complex care, since the rapid and necessary treatments lead to a drastic hormonal change, which is abrupt, severe, and unexpected by the patients. The hormonal changes critically subvert the hormonal assets, leading to psychological and physical effects such as a still not well-known systemic dryness. This breast cancer iatrogenic dryness (BCID) may be related: (1) to the functional hyperandrogenism, apparently similar to Polycystic Ovary Syndrome (PCOS), affecting young women; (2) to the pharmacologically induced menopause by aromatase inhibitors (AIs), which causes a two-fold increase in dryness symptoms compared to untreated women; and (3) to a long-term perioperative chemotherapy, which frequently is mandatory [17].

Recently, some authors focused on the association between dry eye syndrome and breast cancer patients, particularly those using AIs [18]. This could be attributed to the decreased aromatization of the A-cycle of steroids, which leads to the conversion of androgens into estrogens, decreasing the extra-ovarian estrogen production and causing sex hormone imbalance [18]. AIs are also increasingly used for the treatment of postmenopausal patients with estrogen/progesterone receptor-positive breast cancer, in subsequent metastatic settings, and as a tool of chemoprevention in women at increased risk of breast cancer [19]. Hence, a comprehensive assessment of one’s sex hormone dynamics during cancer treatment and appropriate interventional measures may be essential in minimizing ocular and overall mucosal side effects.

This review aims to describe the impact of breast cancer surgery on women’s hormonal setting, by understanding the relationship between physiological sex hormone dynamics and dry eye pathophysiology in similar sex hormone-dependent diseases.

## 2. Materials and Methods

A literature review of articles on the role of sex hormones in dry eye syndrome and systemic hormonal replacement therapy that were published between March 1974 and March 2021 available on PubMed, ClinicalTrial.gov, and Web of Science was carried out without any restriction of language. All published peer-reviewed randomized clinical trials, meta-analysis, systematic reviews and observational studies about ocular surface disease related to sex hormone imbalance (i.e., breast cancer treatment, PCOS, menopause) were evaluated. A total of 95 manuscripts were included.

Patients’ written informed consent was obtained for each of the included images.

## 3. Results

### 3.1. Pathogenesis

#### 3.1.1. Role of Sex Hormone Balance on the Ocular Surface Fitness

Estrogens exert immunoenhancing activities and stimulate an immune response by inducing synthesis of antibodies and by triggering cell-mediated inflammation through the activation of the ERα, while androgens may have a suppressive effect on the humoral and cellular immune response, and function as natural anti-inflammatory modulator [20,21,22].

However, in breaking one’s immune tolerance, disturbances in estrogens, androgens and DHEA could affect the target tissue or the immune-inflammatory effector cells or both [23]. Therefore, it is assumed that sex steroid imbalance primarily predisposes to inflammation. Moreover, it has been widely accepted that sex steroid imbalance induces autoimmunity in genetically predisposed individuals. This genetic predisposition might regulate target sensitivity to sex steroid imbalance and/or the host responsiveness against the changes and cellular remnants produced in the target tissue.

These primary data suggest that steroid hormones may play an important role in maintaining the ocular surface equilibrium and function [24]. Although the precise mechanisms are not yet clear, steroid hormones may contribute to the regulation of the function of tear film and the maintenance of homeostasis at the ocular surface [25]. Notably, sex hormone levels may modulate aqueous tear production through their effects on the ocular surface [26]. Moreover, some studies showed that dry eye syndrome (DES) symptoms, identified with the ocular surface disease index (OSDI) questionnaire, worsened with systemic hormonal replacement treatments (SHRT) using drospirenone and estradiol, but Schirmer test and tear film break-up time (TF-BUT) improved after 6 months of treatment. In fact, it has been shown that low estrogen concentrations tend to induce acinar cell apoptosis in climacteric women. Sex hormone effects in target cells might also precipitate and direct immune-inflammatory attacks to affected cells, exposing female breast surgery recipients to a higher risk of developing autoimmune disease in the long term. 

**Figure 1:**
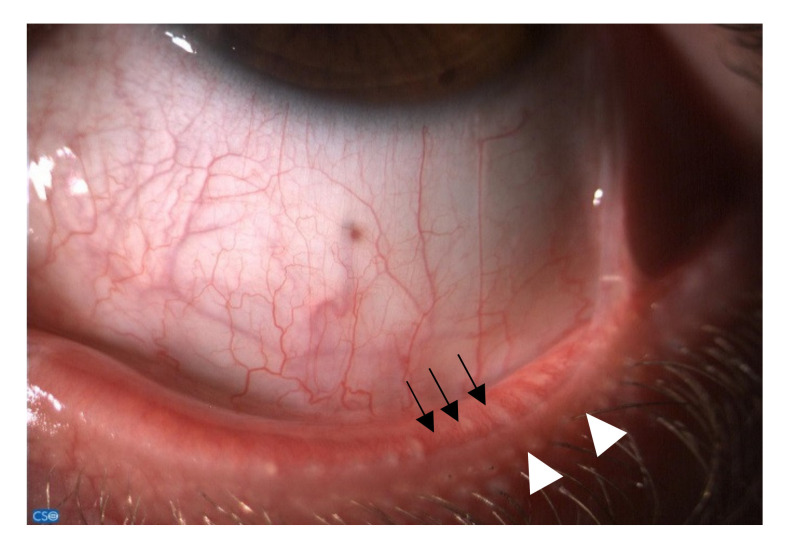
Dysfunction of the meibomian glands (white arrowheads) and telangiectasia (black arrows) on the edge of the lower eyelid.

The alteration of meibomian gland function and morphology by sex hormones is noteworthy, as it may be implicated in numerous dry eye-related ocular surface diseases (Figure 1) [27]. As a consequence, decreased local androgen to estrogen ratio induced by even physiologic changes, pathologies or treatments—such as menopause, autoimmune diseases, anti-androgen treatments, and SHRT—may cause meibomian duct obstruction via epithelial hyperkeratinization [27]. This, ultimately, disrupts the tear film stability, increasing vulnerability to dry eye disease. Interestingly, meibomian glands innately express machineries to locally synthesize sex hormones, suggested by the glandular cell expression of the mRNAs of all necessary steroidogenic enzymes [28].

However, it should be noted that there have been conflicting reports between serum sex hormone levels and the incidence of dry eye in postmenopausal women, warning that the former may not be fully indicative of clinical symptoms [29,30,31]. Although unclear, this could be partly attributed to the greater importance of the peripheral conversion of sex hormones compared to circulating levels, as well as feedback mechanisms from gland dysfunction causing increased release of sex hormones [29].

#### 3.1.2. Estrogens

The role of estrogens in dry eye is not well defined, with apparently contradictory effects in different tissues of the ocular surface and at different circulating estrogen levels. Animal evidence and human in vitro studies suggest that estrogens inhibit meibomian gland secretion, where they may also promote inflammation. Estrogens have been found to reduce the size and lipid output of sebaceous glands, to influence lipid secretion by inhibiting lipogenesis, and to promote inflammatory response on the ocular surface, 17-β-estradiol in particular. A possible pathway of estrogenic effects on meibomian glands could be through suppression of androgen-induced glandular activities by competitive binding to androgen receptors and/or by their initial transcription [29,32]. Additionally, estradiol may downregulate cyclic AMP signaling in meibomian gland epithelial cells, leading to cellular proliferation [33]. Its effect on corneal epithelium and in the lacrimal gland is unclear; it can have both a proinflammatory and an anti-inflammatory effect [34,35].

Clinical evidence is similarly inconclusive. Both higher and lower circulating estrogen levels in postmenopausal women have been associated with reduced tear function [36]. However, the impairment of several functions at the ocular surface appears to be related to the estrogen peak occurring during the follicular phase of the menstrual cycle, especially in dry eye patients. In this group of patients, the concurrent chronic inflammation leading to aggravation of ocular dryness may strengthen the evidence for estrogen’s role in the upregulation of proinflammatory products in tears.

Although its direct contribution to sex hormone-related dry eye is unclear, meibomian gland morphology has been shown to be affected by estrogen and progesterone in murine models. In an ovariectomized mouse model, treatments with estrogen, progesterone, or both produced unique alterations to the structure of the meibomian gland [37]. More importantly, similar effects were not observed with androgens, or a lack thereof, suggesting that estrogenic effects on meibomian gland morphology is a unique feature. It is important to note that hormonal changes, as well as systemic hormonal treatments such as estrogen replacement therapy in women, may contribute to the etiology of meibomian gland dysfunction (MGD) and anterior blepharitis.

#### 3.1.3. Androgens

On the contrary, effects of androgen on the ocular surface seem relatively clear. Androgens exert a pro-sebaceous effect on meibomian glands, and have been found to suppress genes associated with keratinization, stimulate genes associated with lipogenesis, and influence the maturation of acinar cells leading to increased lipid secretion. Similarly, androgen deficiency, androgen receptor dysfunction and insensitivity, and the use of anti-androgen medications have been shown to be associated with obstructive MGD.

Although the meibomian glands contain both androgen and estrogen receptors, androgens are believed to play the main role in tear production, due to their positive effect on the lipid quality and quantity produced by meibomian glands [38,39,40,41,42,43]. Multiple key aspects of lipogenesis have been shown to be directly affected by androgens in meibomian glands, through the upregulation in mouse models of critical lipogenic enzymes, including adenosine triphosphate (ATP)-citrate lyase, acetyl-CoA synthase, acetyl-CoA carboxylase, acetoacetyl-CoA synthase, and 3-hydroxy-3-methylglutaryl CoA synthase 1 [41].

These effects are clinically confirmed by alterations to the meibomian glands observed in patients affected by complete androgen insensitivity syndrome (CAIS). Lack of androgen response showed a significant increase in meibomian gland orifice metaplasia, eyelid keratinization, telangiectasia and erythema, along with a decrease in tear meniscus quality [38]. Moreover, even the increase in signs and symptoms of dry eye in patients undergoing antiandrogenic treatments for prostatic diseases is indicative of androgen function on tear production [44]. In addition, women with Sjogren’s syndrome have been shown to have reduced serum androgen levels, not attributed to OCPs and SHRT, partially explaining the pathogenesis of dry eye in these patients [45].

#### 3.1.4. Breast Cancer Treatment and Dry Eye Syndrome

Breast cancer is the most common tumor among females. 2.2 million cases were reported in 2020, which means that almost 1 woman in 12 develops breast cancer in her lifetime [46].

Additionally, hormone receptor–positive breast carcinoma is the most frequent subtype of breast cancer around the world [47].

In particular, estrogen receptor alpha-positive (ERα+) subtype of breast cancer (BC) makes up approximately 75% of all diagnoses. About half of all ERα+ BCs are also positive for progesterone receptors (PgR+), whose gene is under the transcriptional control of ERα and its ligands (e.g., estradiol or E2). ERα+/PgR+ BC is also referred to as HR-positive BC [48]. The treatment of women with HR+ BCs often includes AI or selective estrogen receptor modulator, such as tamoxifen [49,50]. Therefore, a high number of women treated for BC receive antiestrogen therapy, specifically AIs, as part of their management regimen.

Breast cancer surgery and perioperative treatments critically affect the normal female physiology [51]. Homeostatic mechanisms which aim to preserve equilibrium are dysregulated by the sudden hormonal break and forced early menopause [25,52] due to the surgery itself, as well as to the post-operative treatments. The ocular surface, under such challenging conditions, also fails to maintain physiologic equilibrium, leading to long-term, frequently persistent ocular discomfort and chronic inflammation [53].

The sex hormone-dependent loss of homeostatic para-inflammatory mechanisms [24] is also enhanced by the repeated lymphoepithelial injuries induced by required radio- and chemotherapy. Together, these postoperative treatments have a synergic detrimental effect, where the loss of restorative capabilities of the ocular surface equilibrium after environmental impacts and sex hormone dysregulation is further compounded by the toxic drugs acting on highly-regenerative ocular surface epithelia.

#### 3.1.5. The Role of Systemic Hormones Therapy on Ocular Surface

Several studies report the impact of sex hormones on the ocular surface, particularly in patients treated with systemic hormonal replacement therapy (SHRT), which has also been proposed as an interesting strategy to improve the ocular surface and tear functions in DES [54,55,56,57].

Schaumberg et al. [58] reported that women receiving SHRT with estrogen and estrogen plus medroxyprogesterone acetate were at increased risk of DES. A possible explanation for these conflicting conclusions is that the outcome of SHRT depends on estrogen dosage and the age of the individuals when therapy is first initiated. Estrogen may be only beneficial in younger women, as the typical age group of breast surgery recipients, whereas it may be detrimental and/or pro-inflammatory in older females [26]. Clinical evidence suggests that estrogen supplementation either improves dry eye symptoms and tear function or has minimal effects [34]. Despite the lack of a definitive effect of external estrogen, it is likely that changes in hormonal balance ultimately influence the function of various sebaceous glands, including meibomian glands.

### 3.2. Functional Hyperandrogenism on the Ocular Surface

When investigating the link between breast surgery and DES, the inherent issue at hand is a potential sex-steroid imbalance and the functional severe hyperandrogenism. However, it remains to be clarified how estrogen or androgen insufficiency or a complex interplay between them serves to increase or protect from dry eye disease. One way to examine this relationship between sex hormone imbalance and dry eye disease is to conduct observational studies in clinical situations where this takes place. Two such examples are patients suffering from polycystic ovary syndrome (PCOS) and patients undergoing menopause. In both conditions, patients experience a relative reduction in blood estrogen and progesterone.

#### 3.2.1. PCOS

PCOS is the most common endocrine disorder in reproductive-age women. The clinical features of PCOS are hyperandrogenism, anovulation, and metabolic syndrome. Associated diseases include type 2 diabetes, obstructive sleep apnea, and depression. Risk factors include family history, obesity, and lack of physical exercise. The potential association between hyperandrogenism and dry eye in PCOS was first reported by Bonini et al. in 2007 [12]. In a prospective observational case series, the team demonstrated that 16 PCOS patients, with both clinical and biochemical signs of hyperandrogenism, had significantly higher rates of dry eye symptoms and objective signs compared to 46 patients with ultrasound-proven polycystic ovary without evidence of hyperandrogenism. The findings regarding the higher rates of symptomatic dry eye disease in PCOS patients than controls were subsequently confirmed by Yavas et al. in 2008, Coksuer et al. in 2011, and Gonen et al. in 2013, with all three studies reporting a higher prevalence of evaporative dry eye in PCOS patients [22,59,60]. Coksuer et al. and Gonen et al. further demonstrated that there were no significant differences in aqueous tear secretion, via Shirmer I test, between PCOS patients and controls [22,60]. A study by Yuksel et al. in 2015, comparing dry eye symptoms and signs in 35 PCOS patients with biochemically proven hyperandrogenism with 27 healthy controls, also found significantly lower TF-BUT in PCOS patients [61].

However, unlike other studies, Yuksei et al. found no significant differences between groups in terms of dry eye symptoms. A proposed underlying mechanism for the higher prevalence of evaporative-type dry eye in PCOS was suggested by Baser et al., who noted that the PCOS group had a significantly higher prevalence of MGD compared to controls [62]. However, it is important to note that the diagnostic criteria for MGD used for this study were based mostly on the presence of posterior blepharitis. No objective assessment or grading of meibomian gland expression and meibum content was reported for the study, making the confirmation of MGD relatively subjective. Another proposed mechanism was highlighted by a study by Asfuroğlu et al., who noted a correlation between subclinical systemic inflammation and dry eye severity in patients with PCOS, as determined by the blood neutrophil to lymphocyte ratio (NLR) and TF-BUT, respectively [54]. It is important to note, however, that while NLR may have proven prognostic utility in certain cancers and infections, its usefulness in otherwise healthy patients is not yet established.

Overall, looking at the data from published research, it appears that DES prevalence is high in PCOS patients, and is linked to hyperandrogenism. Furthermore, the type of dry eye that is most prevalent in this cohort is evaporative-type dry eye. However, the underlying mechanism will require further studies, particularly those looking into ocular surface inflammation measured by tear cytokines and impression cytology, as well as tear lipid content determined by lipidomic studies on expressed meibum. It is also important to consider the potential confounding factors and effects of diabetes and obstructive sleep apnea on dry eye prevalence and severity in PCOS patients. Such conditions are independently known to increase the risk of dry eye disease. Nevertheless, the aggregated results on PCOS and dry eye provide a useful model to determine the potential effects of breast cancer treatment on dry eye symptoms and signs.

#### 3.2.2. Menopause

Menopause is the permanent cessation of menstruation in women. While the most significant change in menopause is a drop in blood estrogen and progesterone levels, it is also important to note that androgen levels decrease progressively as well. There is strong evidence that menopause is associated with an increased risk of dry eye disease [55,63]. Similar to PCOS, the type of dry eye disease is predominantly evaporative in nature, with an association with MGD [58,64]. Furthermore, Ziemanski et al. demonstrated, in a prospective observational study on dietary habits in postmenopausal women with dry eye disease, that high omega-3 and moderate omega-6 consumption were associated with significantly lower risks of MGD [56].

Despite the significant drop of estrogen and progesterone levels, it may not be the major underlying reason for dry eye in menopausal patients. A meta-analysis by Dang et al. showed no significant improvements in dry eye symptoms for patients taking SHRT for menopausal symptoms [57]. A meta-analysis by Liu et al. concurred with this finding, but noted that SHRT therapy did increase Schirmer test scores in treated patients compared to controls, despite having no impact on OSDI score and TF-BUT [65]. A prospective clinical trial by Feng et al. demonstrated the effectiveness of SHRT in ameliorating dry eye symptoms and signs only in perimenopausal patients less than 50 years of age [66]. Therefore, it appears that SHRT may be more effective earlier when estrogen and progesterone levels have not yet bottomed out.

In fact, for postmenopausal women, SHRT seems to stimulate adverse effects on the ocular surface that lead to dry eye disease. A study showed that the longer the duration of SHRT use in postmenopausal women, the higher increased the risk of dry eye disease [36]. The possible lack of efficacy of SHRT on dry eye disease may be partially attributed to a lack of robust large-scale randomized controlled trials on the subject.

An alternative explanation, one that has received strong support from the scientific community, is that MGD and dry eye disease in menopause are a result of the reduction in androgen, instead. This is supported by the work of Ablamowicz et al., in which the severity of measured meibomian gland dropout correlated with blood testosterone levels in postmenopausal women [30]. This is further supported by the beneficial effects of androgen replacement therapies on dry eye signs and symptoms in patients with significantly low testosterone levels [42]. It is, however, difficult to separate this observation from the confounding effect of the aging meibomian glands and their atrophy. As such, the underlying mechanism for menopause-induced dry eye disease remains unclear. Rather than treating menopausal dry eye with SHRT, an alternative is to consider local hormonal therapy in the form of eyedrops directly applied to the ocular surface. However, results from preliminary studies using this mode of drug delivery are conflicting [25].

In summary, the two conditions—PCOS and menopause—are consistently associated with an increased risk of evaporative dry eye disease. While both conditions are characterized by a reduction in estrogen and progesterone, PCOS exhibits hyperandrogenism, while menopause exhibits hypoandrogenism. Clearly, neither the absolute hormonal level alone nor the interplay between sex hormones explains the mechanism for dry eye disease in these cohorts. To understand the link between sex hormone interaction and dry eye disease, we need to consider the additional confounders that will inevitably play a part in dry eye pathogenesis.

### 3.3. Diagnosis and Clinical Aspects

**Figure 2:**
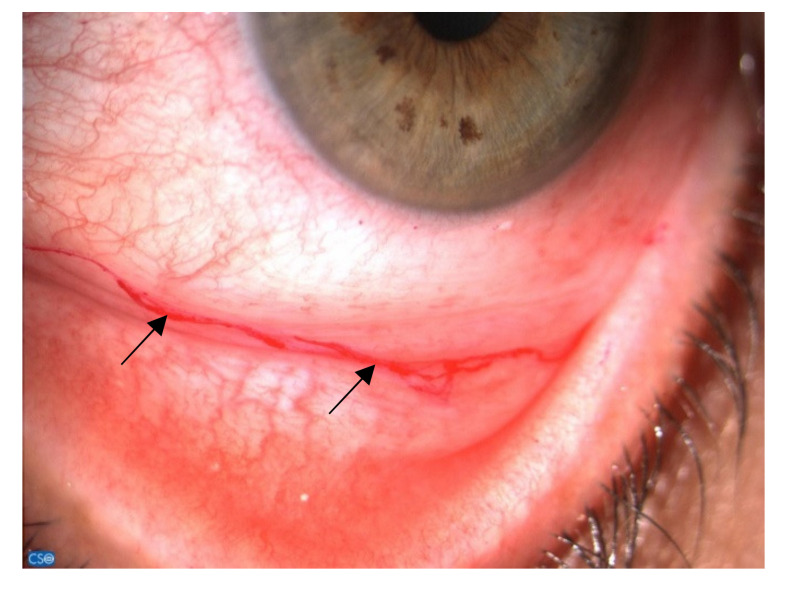
Rose bengal-colored mucus filaments (black arrows) in the lower fornix.

Although validated protocols are not yet available, administration of a subjective symptom questionnaire along with clinical evaluation of the ocular surface system is usually recommended among patients receiving more than four cycles of chemotherapy or targeted therapy [67]. Ocular surface findings in breast cancer patients are not particularly different from those of patients with evaporative dry eye, although those women suffer from severe itching along with ocular discomfort and inflammation, and usually show a severe mucus secretion, frequently leading to a mucus fishing syndrome (Figure 2). Finally, along with the tear film instability and meibomian gland dysfunction, those patients usually are intolerant to the use of contact lenses. Considering that the relationship between signs and symptoms of dry eye disease is not linear, and varies across individuals and dry eye disease subtypes, the ability to accurately quantify ocular symptoms is an important screening tool for establishing the need for additional evaluations [68].

Different patient-reported outcome questionnaires have been validated for this task in the clinical setting. Among these, Ocular Surface Disease Index (OSDI) is a validated 12-item polytomous response questionnaire, where a score over 12 is indicative of dry eye disease [69]. In addition, patients with positive symptoms can be stratified as mild, moderate or severe dry eye disease according to the final score (respectively, 12.1–22, 22.1–32 and >32.1).

Instead, tear film instability is evaluable in different ways. The most frequently employed test is the measurement of fluorescein TF-BUT, represented by the time interval between a complete blink and the appearance of the first black spot in the tear film. The reference value for DES diagnosis when fluorescein is used ranges from a cut-off time of less than 10 s, to less than 5 s when smaller, more controlled volumes of fluorescein are used [68]. Since tear film stability can be affected by fluorescein itself, non-invasive all-in-one devices for TF-BUT measurement have been developed and have fueled its popularity [70].

Among dry eye disease tests, tear osmolarity has been shown to have the highest correlation with disease severity, and was frequently reported as the best single metric to diagnose and classify dry eye disease [71]. Indeed, mean osmolarity values usually increase with disease severity, classified as normal (302.2 ± 8.3 mOsm/L), mild-to-moderate (315.0 ± 11.4 mOsm/L) and severe (336.4 ± 22.3 mOsm/L). Furthermore, increased variability between eyes and between visits is considered another index of severity [72]. In particular, between-eye differences beyond the threshold of 8 mOsm/L should be considered an indication of the loss of tear film homeostasis that occurs with DES [68].

Tear film volume is an important determining factor of dry eye disease, which may be both a key pathologic component and a diagnostic sign of the disease. Traditionally, tear production is measured by a Schirmer test, which measures the distance traveled by a tear on a paper strip placed in the inner lower eyelid after a period of 5 min. However, several cut-off values have been proposed (from <5 mm/5 min to <10 mm/5 min) with different ranges of sensitivity and specificity. However, in our patient population, tear film secretion is usually within normal limits.

**Figure 3:**
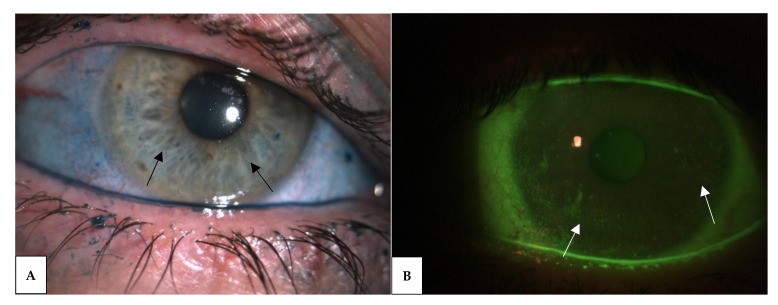
Punctate epithelial keratopathy (black and white arrows) highlighted by staining with Lissamine Green (image **A**) and Sodium Fluorescein (image **B**) in the same patient’s eye.

The staining of ocular surface epithelium is currently used for diagnosis, classification and characterization of dry eye disease. Sodium Fluorescein and Lissamine Green are the two most commonly used dyes that are able to stain the cornea and the conjunctiva, respectively (Figure 3), so they are critical in assessing BCID severity as well as therapeutic and prognostic biomarkers of the BCID. Examination of eyelid features is also crucial for dry eye subtyping. It should include inspection of the eyelashes for anterior blepharitis or demodex infestation and eyelid margin and meibomian gland orifices for MGD. Infrared meibography allows the observation of the meibomian gland structure and provides useful parameters, such as dropout, tortuosity, and vagueness [73].

Corneal sensitivity, assessable with Cochet-Bonnet or non-contact air-jet esthesiometers, and conjunctival hyperemia score can also be measured to better understand treatment response or disease severity [74,75]. In particular, conjunctival redness is the most common clinical sign, suggestive of ocular surface inflammation, which is easily detectable with standard slit lamp biomicroscopic examination [68].

Finally, conjunctival impression cytology and tear sampling may help in the assessment of the local inflammation status, by analyzing cellular bio-markers such as matrix metalloproteinases, cytokines, chemokines and HLA-DR. Moreover, in such patients, the reported changes in mucins tear secretion and cell surface expression can be unveiled by immunostaining of cytological specimens [68].

### 3.4. Therapy

Fear of cancer recurrence is not the only thing that affects the quality of life of breast cancer survivors, because managing therapy side effects is also challenging [51].

The impact of drugs varies widely, and their effect may be altered by the ambient estrogenic and androgenic milieu at the time of initiation.

Treatment-related physical changes lead to a negative effect on one’s body image, along with the potential loss of erogenous sensations of the breast or genitals. Fatigue, insomnia, depression, and anxiety, as well as partner issues, are contributing factors beyond physical and hormonal changes. For many women, however, the consequences of iatrogenic menopause or estrogen deprivation therapy have the greatest negative impact on sexual function [76].

Hormone deprivation syndrome is characterized by vasomotor symptoms, genitourinary symptoms, and sexual health concerns, such as vaginal atrophy, dyspareunia, and the inability to have penetrative vaginal intercourse [77].

Risk-reducing bilateral oophorectomy, with or without hysterectomy, also has a profound effect on sexual function due to the onset of immediate and untreated menopausal symptoms. Additionally, oophorectomy results in the loss of secreted ovarian androgens beyond the expected drop from natural menopause [78].

Since ocular toxicities induced by anti-cancer agents, such as dry eye disease, are not preventable in breast cancer patients, clinicians must be aware of these potential complications. In fact, timely diagnosis and intervention can lead to better quality of life in patients undergoing systemic adjuvant therapy, thereby ensuring patients’ compliance with anti-cancer treatment.

Even though several studies show that exogenous androgens are promising as treatment options for dry eye disease, the increase in systemic circulating levels of steroid hormones has relevant side effects; therefore, local administration of androgens has been considered by clinicians as a treatment for dry eye disease [79,80]. Two notable examples are local administration of androgens through eye drops or through transdermal patches on eyelids, showing beneficial effects in dry eye patients. A National Institute of Health (NIH) study showed that 30% of dry eye patients became asymptomatic after treatment with androgen eye drops, compared with 8% of controls [81]. The transdermal patches, on the other hand, showed a 51% decrease in dry eye symptoms, with the added benefit of reduced irritability caused by the poor solubility of androgen eye drops [82].

On the contrary, exogenous estrogens have generally been shown to have deleterious effects on the ocular surface, tear production, and meibomian gland functions by reducing lipid production and gland size [4,29,83,84,85,86,87]. Despite the lack of a clear major mechanism, HRTs and OCPs have been repeatedly reported as risk factors for dry eye disease [88,89,90,91]. In a large-scale cohort study of 25,665 postmenopausal women with 48-month follow-up, a 69% higher prevalence of dry eye disease was observed among postmenopausal women using estrogen-only HRT compared to controls, with slightly lower prevalence (29%) among those using estrogen and progesterone treatment [88].

However, unlike consistent evidence on the effect of testosterone on dry eye and meibomian glands, reports on various estrogen sources—such as OCPs and menstrual cycle dynamics—have shown conflicting results. In the 2017 Tear Film and Ocular Surface Society Dry Eye Workshop (TFOS DEWS) report, systemic hormonal therapies and HRT were referred to as drugs with associated risk of dry eye disease [90]. Another study by Chen et al. reported an increase in OSDI scores among subjects who used OCPs and contact lenses concurrently in the previous 30 days, compared with those who did not use any of the above, implying that OCPs could affect contact lens tolerance and vulnerability to dry eye [92]. On the other hand, comparisons between follicular phase and premenopausal luteal phase on osmolality and tear volume, evaporation rate and turnover rate were not significant, with similar results reported between OCP users and controls [93]. Furthermore, although they were from a decades-old study, the results of Schirmer type I and tear break time were not significantly different between women using OCPs and non-users [94]. These results together likely suggest that the change in androgen dynamics and hormonal balance may be the main modulator of meibomian gland function, and that estrogen is a secondary modulator which acts primarily as a dampener of androgenic effects [95].

Even if estrogen levels show a conflicting correlation with dry eye disease, a distinctive role of estrogen in dry eye development has been shown in murine models. A unique effect of estrogen, as well as progesterone, is the alteration of the morphology of the meibomian gland. Indeed, in an ovariectomized mouse model, treatments with estrogen, progesterone and estrogen plus progesterone produced unique alterations to the structure of the meibomian gland [37]. Interestingly, androgen treatments in orchiectomized mice and mice with androgen receptor dysfunction did not show the same effect. Additionally, 17β-estradiol resulted in reduced lipid synthesis and increased lipid catabolism in ovariectomized mice, linking meibomian gland dysfunction and evaporative dry eye with increased estrogen [32]. This negative effect on tear film stability and lipid production could explain how exogenous estrogens (i.e., OCPs) cause susceptibility to evaporative dry eye and impaired tolerance to contact lens usage [92]. Although the specific interaction and synergistic effects of estrogen and progesterone are still under investigation, a reduced prevalence of dry eye disease in patients using estrogen and progesterone HRT, compared to those using estrogen-only HRT, suggests that progesterone may partially counteract the effects of estrogen on the meibomian glands and on the composition of the tear film [88].

## 4. Discussion

In addition to fighting this terrible disease, patients must face several systemic side effects induced by iatrogenic menopause and by estrogen deprivation therapy, as well as perioperative chemotherapy, with important psychological implications. Among these, BCID and dry eye disease, in particular, are life-threatening complications that critically affect women’s daily life, beyond their complex fighting against cancer. This status may be due to a loss or failure of immune regulatory mechanisms, which usually maintain homeostasis by para-inflammation [96,97], subsequent to the hormonal changes, such as in other ocular diseases [24,98]. The BCID pathogenesis is also associated with a systemic estrogen-level drop, and a consequent functional hyperandrogenism, as in case of PCOS, and, rarely, with an unexpected hypoandrogenism, as in case of physiological menopause. Thus, these women after breast surgery experience a severe chronic evaporative dry eye syndrome with mucus filaments and frequent intolerance to wearing contact lenses. Most of them suffer from moderate itching associated with the usual ocular discomfort caused by the dryness.

Such hormonal changes also occur in other phases of women’s daily life, such as menopause and the menstrual cycle, as well as in other pathologic conditions such as PCOS and functional hyperandrogenism. In these conditions, an overall reduction in mucosal tissue lubrification and increased mucus production are related to a decrease in the tear aqueous component and an alteration in ocular surface mucin production [12]. Moreover, sex hormones may influence ocular surface-modulating immune response, leading to a low-grade subclinical inflammation, caused by the subverted para-inflammatory mechanisms that maintain ocular surface equilibrium. However, although sex hormone alteration may influence ocular surface status, PCOS and menopause studies have taught us that, rather than a specific activity of estrogens and androgens, it is above all the homeostatic balance between them that guarantees the normal function of the ocular surface. Therefore, an imbalance of sex hormones induces a dysregulation of the innate para-inflammatory response [24], which causes the failure of the ocular surface and overall mucosal dryness.

Despite the multifactorial nature of BCID, meibomian gland physiology is most likely the single major bridge between sex hormones and dry eye disease. Meibomian gland function is balanced between protective androgen effects and androgen-modulating effects of estrogens. Therefore, healthcare providers should be mindful, in the current state of research, that androgens are the main modulator of meibomian gland function and sex hormone-related dry eye. Assessment of androgen levels in dry eye disease patients should be prioritized for patients with sexual hormone imbalances, such as menopause. Before setting up sex hormone-altering treatments, physicians should ideally assess patients’ risk factors for dry eye disease; however, these therapies are often necessary to relieve more severe pathologies and symptoms. Therefore, initiating estrogenic or anti-androgenic treatments may be inevitable, despite the high risk of dry eye disease. In these cases of induced dry eye disease, clinicians should first consider the aforementioned local androgen administration or estrogen-progesterone HRTs for postmenopausal women looking for treatment options. Breast cancer is the most common cancer among females and it has devasting consequences in patients’ lives. Medical treatment and surgery related physical changes lead to a negative effect on one’s body image, depression and anxiety, as well as partner issues related to physical and hormonal changes. For many women, the consequences of iatrogenic menopause or estrogen deprivation therapy have the greatest negative impact on sexual function [76].

However, the ethology of this clinical entity is still controversial, as well as the ocular signs and symptoms. Therefore, the BCID ocular management is still mainly focused on the ocular surface training by lid hygiene, warm compresses and lipophilic artificial tears, as well as by oral omega −3 and −6 supplementation. In fact, the early use of hormonal topical or systemic treatment has been not fully beneficial in improving dry eye disease, and it is still not completely safe in such post-cancer patients.

## 5. Conclusions

Breast cancer iatrogenic dryness is a systemic condition that is a consequence of the hormonal changes caused by necessary tumor treatments. The ocular involvement with severe dry eye, as well as the overall mucosal dryness, critically limits the daily life choice and activity of such women, therefore should be taken into account in the medical management of this large group of patients.
